# Effects of Multiple Sedentary Days on Metabolic Risk Factors in Free-Living Conditions: Lessons Learned and Future Recommendations

**DOI:** 10.3389/fphys.2016.00616

**Published:** 2016-12-09

**Authors:** Teatske M. Altenburg, Joost Rotteveel, Erik H. Serné, Mai J. M. Chinapaw

**Affiliations:** ^1^Department of Public and Occupational Health, EMGO Institute for Health and Care Research, VU University Medical CenterAmsterdam, Netherlands; ^2^Department of Pediatrics, VU University Medical CenterAmsterdam, Netherlands; ^3^Department of Internal Medicine, VU University Medical CenterAmsterdam, Netherlands

**Keywords:** sedentary lifestyle, uninterrupted sitting, metabolic risk, postprandial, healthy adults

## Abstract

**Background:** Recent experimental studies in adults have demonstrated that interruptions to prolonged sitting have beneficial effects on metabolic risk factors in adults, compared to prolonged sitting. We explored the hypothesis that multiple consecutive days of predominantly prolonged sedentary time may have an unfavorable effect on the postprandial response of C-peptide, glucose, and triglycerides in free-living healthy young men.

**Methods:** In this explorative pilot study, healthy young men (*n* = 7; 18–23 years) consumed standardized mixed meals at 1 and 5 h during two experimental laboratory-sitting days, with 6 days of predominantly prolonged sedentary time in between. Serum and plasma samples were obtained hourly from 0 to 8 h for measurement of glucose, C-peptide, and triglycerides. Participant's sedentary time was monitored using an accelerometer during the prolonged sedentary days as well as during 6 normal days prior to the first laboratory day. Differences in postprandial levels were assessed using generalized estimating equations analysis. Due to the explorative nature of this study and the small sample size, *p*-value was set at <0.10.

**Results:** Overall, when expressed as % of wear time, sedentary time was 5% higher during the 6 prolonged sedentary days, which was not significantly different compared to the 6 normal days (*n* = 4). Following 6 prolonged sedentary days, postprandial levels of C-peptide were significantly higher than at baseline (B = 0.11; 90%CI = [0.002; 0.22]; *n* = 7). Postprandial levels of glucose and triglycerides were not significantly different between the 2 laboratory days.

**Conclusions:** Due to the relatively high sedentary time at baseline, participants were unable to increase their sedentary time substantially. Nevertheless, postprandial C-peptide levels were slightly higher after 6 prolonged sedentary days than after 6 normal days.

## Introduction

Accumulating evidence from population-based studies indicates that prolonged sitting may have negative health effects in adults (Healy et al., 2008; Henson et al., 2013). Experimental studies on the acute effects of prolonged sitting in overweight/obese (Dunstan et al., 2012) and healthy (Peddie et al., 2013; Bailey and Locke, 2015) adults demonstrated that 1 day of uninterrupted sitting resulted in significant higher postprandial glucose and insulin levels, when compared to brief walking interruptions, i.e., 2 min interruptions every 20 min (Dunstan et al., 2012; Bailey and Locke, 2015) and 1 min 40 s interruptions every 30 min (Peddie et al., 2013), during prolonged sitting. However, brief standing interruptions, i.e., 2 min every 20 and 30 min, did not lower postprandial glucose and insulin levels in healthy adults (Miyashita et al., 2013; Bailey and Locke, 2015). Additionally, Altenburg et al. (2013) showed that 1 day of uninterrupted sitting resulted in significantly higher postprandial levels of C-peptide [i.e., reflecting endogenous insulin (Polonsky and Rubenstein, 1984; Van Cauter et al., 1992)] in healthy young men, when compared to hourly 8-min moderate-intensity physical activity interruptions to sitting.

Two recent studies compared the metabolic effects of sustained days of prolonged sitting in overweight/obese adults with sustained days of reduced (Thorp et al., 2014) and interrupted (Larsen et al., 2015) sitting in a laboratory setting. In a 3-day randomized crossover study Thorp et al. (2014) demonstrated that postprandial glucose level, but not insulin and triglyceride levels, was higher over the course of a day during prolonged sitting compared to a day of alternate standing and sitting in 30-min bouts. No temporal changes (day 1 vs. 5) were found in this study (Thorp et al., 2014). Similarly, in a 3-day randomized crossover study Larsen et al. (2015) found that sustained days of prolonged sitting resulted in higher postprandial glucose and insulin levels when compared to days with 2-min light activity interruptions every 20 min (i.e., treadmill walking), but no temporal changes (day 1 vs. 3) were found.

To date, only one study examined sustained days of prolonged sitting in free-living conditions (Lyden et al., 2015). In this study, young and healthy participants (*n* = 10; 4 males) were asked to increase their sitting time as much as possible for 7 consecutive days, limit their standing and walking and refrain from structured exercise and physical activity (Lyden et al., 2015). After 7 days of increased sitting time (i.e., sedentary time increased from 61 to 76% of wear time), glucose concentrations in response to a 2-h glucose tolerance test were similar, whereas insulin concentrations were significantly elevated. The current pilot study is the second study exploring the effects of 6 or more consecutive days of predominantly prolonged sitting in free-living conditions on postprandial glucose and lipid metabolism in healthy young adults. Confirmation of the findings from Lyden et al. (2015) is necessary to gain insight in the potential unfavorable health effects of consecutive days of prolonged sitting in free-living conditions. We hypothesized that 6 consecutive days of predominantly prolonged sitting may have an unfavorable effect on postprandial levels of C-peptide, glucose, and triglycerides in healthy young men.

## Materials and methods

### Participants

Seven males aged 18–23 years participated in this exploratory pilot study. Participants were recruited through distribution of flyers, announcements on University websites and Dutch recruitment websites. Participants were included if they (1) were normal weight (i.e., BMI <25), (2) were apparently healthy, (3) spent at least 30 min/day of moderate-to-vigorous physical activity (MVPA), (4) spent at least 20 min/day of vigorous physical activity on at least 2 days/week, (5) spent on average <2 h/day on prolonged sedentary time, (6) were Dutch or English speaking, and (7) signed a written informed consent. Exclusion criteria were major illness/injury or physical problems. Participants were screened using a health check questionnaire, including questions on medical history (e.g., heart, kidney, joint, muscle, asthmatic complaints; coagulation problems; chest pain) and usual pattern of sedentary behavior and physical activity was screened by dialogue. Participants were requested to refrain from any MVPA for at least 48 h prior to the experiment, and to avoid drinking alcohol and smoking for at least 24 h prior to the laboratory days. Finally, participants were requested to use passive transport (i.e., public transport or car) on the laboratory days.

### Study design and procedures

**Figure 1:**
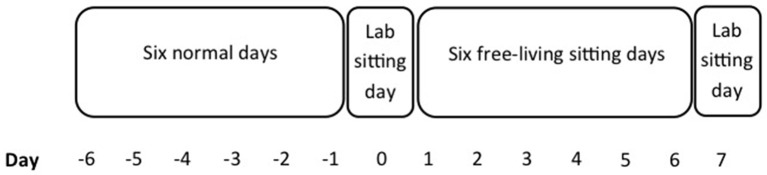
**Study design**.

This explorative pilot study involved 2 experimental laboratory-sitting days (i.e., pre- and post-test), with 6 consecutive increased prolonged sitting days in between (see Figure 1). As sitting time during the 6 consecutive sitting days was measured using accelerometers, these days were referred to as days of increased prolonged sedentary time (i.e., prolonged sedentary days). Accelerometer data of the prolonged sedentary days was used to check for the total (prolonged) sedentary time. Participants also wore an accelerometer during a normal week prior to the first laboratory day, to assess their “regular” daily activity. This study was approved by the Medical Ethics Committee of the VU University Medical Center in Amsterdam (No. 2011/171) and is in accordance with the Declaration of Helsinki.

On the evening before each laboratory-sitting day, participants consumed a standardized meal and snack. On the laboratory sitting days, participants visited the research unit after a 10-h fast. During the first visit, the informed consent and a health history were completed, and baseline anthropometrics were obtained (*t* = 0). Subsequently, an indwelling venous catheter was inserted in the antecubital vein of the left arm, to collect a baseline blood sample and to allow hourly blood sampling during the laboratory-sitting days. Participants then sat quietly for 1 h, in order to achieve a “steady state.” After 1 h, participants consumed a standardized liquid high fat mixed meal, which they were requested to drink within 10 min. Then participants remained seated in a comfortable reclining chair for the next 7 h. They were allowed to use the computer (e.g., watching movies, surfing on the Internet, or reading). Participants were instructed to minimize excessive movement but were allowed to visit the toilet. After 5 h of sitting (*t* = 5), participants consumed a standardized solid high fat mixed meal. Blood samples were collected hourly during each 8-h laboratory-sitting day (i.e., nine blood samples).

### Prolonged sedentary days

During the 6 days of increased prolonged sedentary time participants were requested to increase their sedentary time as much as possible, directing them to remain seated for at least 8 h per day between 7 a.m. and 8 p.m. Participants were requested to spend four of these eight sedentary hours uninterrupted, except for visiting the toilet. During the remaining four sedentary hours, participants were allowed to interrupt their sedentary time once per hour, up to a maximal duration of 15 min, at a light- or moderate-intensity. Since total accumulated sedentary time had to be 8 h, participants had to lengthen (i.e., compensate) their sedentary time for each interruption. Participants were requested to refrain from vigorous physical activity during the 6 prolonged sedentary days.

### Standardized meals

Participants were studied in the postprandial state, as this is more likely to represent “normal daily life” than the fasted state. Importantly, the peaks in glucose and lipids induced by high-calorie (i.e., high carbohydrate and saturated fat content) meals are associated with biochemical inflammation, endothelial dysfunction and sympathetic hyperactivity (Eberly et al., 2003; Ceriello et al., 2004, 2006; O'Keefe and Bell, 2007). When repeated multiple times each day, these peaks in glucose and lipids increase the risk for atherosclerosis and CVD (O'Keefe and Bell, 2007). Maintenance of normal fasting and postprandial glucose levels depends on the ability to create an adequate insulin response to a meal. Participants were requested to consume a standardized meal dinner and an optional snack on the evening before each laboratory-sitting day. The dinner (three choices) consisted of 15 ± 5 g fat, 70 ± 10 g carbohydrates, and 25.8 ± 5.2 g proteins (in total 526.7 ± 40.4 kcal). The snack consisted of 0.6 g fat, 48.6 g carbohydrates, and 2 g proteins (in total 213 kcal). Participants were requested to consume the same meal (and snack) on the evening before each laboratory-sitting day.

The standardized liquid high fat mixed meal given after the first hour of “steady state” sitting (i.e., breakfast) consisted of 58.8 g fat, 92.0 g carbohydrates, and 15.6 g proteins (in total 843 kcal). The standardized solid high fat mixed meal consumed after 5 h of sitting (i.e., lunch) consisted of ~ 77.1 g fat, 116.7 g carbohydrates, and 27.7 g proteins (in total 1190 kcal). The fat, carbohydrate, and protein content of the standardized meals were based on previous studies by our group in young adults, demonstrating postprandial increases in levels of HDL cholesterol, triglyceride, insulin, and glucose (Rotteveel et al., 2008; Altenburg et al., 2013).

### Measurements

Height was measured with a Harpenden stadiometer with an accuracy of 0.1 cm, averaging three measurements. Weight was measured with a calibrated electronic scale (SECA 703) with an accuracy of 0.1 kg. Waist and hip circumference were measured with a flexible band with an accuracy of 0.5 cm. Body fat percentage was measured in a lying position using Bio-electrical Impedance Analysis (Maltron Body Composition Analyzer, BF-906) with an accuracy of 0.1%.

Plasma glucose levels were immediately assessed, within 10 s after collection, using the YSI2300 STAT Plus Analyzer (YSI, Yellow Springs, OH, USA) with an accuracy of 0.2 mmol/l. The second sample was centrifuged (10 min at a frequency of 3000 rpm) and subsequently stored at −80°C. From this sample, C-peptide and triglycerides were determined in heparin gel samples. All samples were analyzed in the same assay. Area under the curve (AUC) and incremental area under the curve (iAUC) were calculated for glucose, C-peptide, and triglyceride using the trapezoidal method, both for the 4- and 7-h postprandial period.

Participants wore an accelerometer (ActiTrainer, ActiLife v5.2.0) for 6 consecutive days, both during the 6 prolonged sedentary days and during 6 normal days before the start of the experiment. Epoch time was set at 15 s to capture the pattern of short duration interruptions to sedentary time. Participants were asked to wear the accelerometer at their right waist (using an elastic belt) during all waking hours, except for water-based activities. Periods of more than 60 min of consecutive zeros were considered as non-wear time and excluded from data analysis. A minimum of 8 h wearing time per day was required to include data in the analysis (Chinapaw et al., 2014).

A cut point of <100 counts per minute (cpm) was selected for overall sedentary time, between 100 and ≤1952 for light physical activity (LPA) and >1952 for MVPA (Freedson et al., 1998). A period of 10 or more consecutive minutes below 100 cpm was defined as a sedentary bout, and time spent sedentary accumulated in sedentary bouts of ≥10 min was defined as prolonged sedentary time (Altenburg and Chinapaw, 2015). To adjust for differences in wear time, overall sedentary time, prolonged sedentary time, LPA, and MVPA time were additionally calculated as relative to wear time.

### Statistics

Descriptive participant characteristics (median [min; max]) were calculated for baseline measures. The blood sample at the end of the first hour of each laboratory-sitting day was considered as steady state and used as baseline blood sample. Related Samples Wilcoxon Signed Rank Tests were used to test for baseline differences in blood levels between the first and the second laboratory day, and to test for differences in accelerometer-derived data (i.e., sedentary time, LPA time, and MVPA time expressed as percentage of wear time) during 6 normal days and 6 increased sedentary days. Generalized Estimating Equations (GEE; univariate) were used to assess the difference in blood levels between both laboratory-sitting days. This longitudinal analysis technique was used to correct for dependency of measures within each participant. All statistical procedures were performed using SPSS software (version 22.0.0). Due to the small sample size and explorative nature of our study we considered a *p*-value below 0.10 as statistically significant.

## Results

Table 1 shows baseline participant characteristics and metabolic risk factors at the start of each laboratory day. Steady state blood values for glucose, C-peptide and triglycerides were not different between the two laboratory-sitting days.

**Table 1:** **Descriptive participant characteristics (mean ± ***SD***; ***n*** = 7)**.

	**Baseline anthropometrics**		**Differences^a^ (*p*-value)**
Age (years)	21.4 ± 2.3		
Height (cm)	183.2 ± 9.2		
Weight (kg)	72.9 ± 2.3		
BMI	21.8 ± 1.4		
Waist/hip	0.9 ± 0.1		
Body fat (%)	13.9 ± 5.2		
**Blood measurements**	**Steady state 1st laboratory day (pre-test)**	**Steady state 2nd laboratory day (post-test)**	
Glucose (mmol/l)	4.5 ± 0.3	4.3 ± 0.1	0.31
C-peptide (mmol/l)	0.35 ± 0.10	0.35 ± 0.08	0.61
Triglycerides (mmol/l)	0.86 ± 0.28	0.99 ± 0.19	0.18
**Accelerometer-derived data**	**Normal days^#^**	**Increased sedentary days^#^**	
	**Median [min; max]**	**Median [min; max]**	
Total wear time (min/day)	765 [668; 863]	882 [713; 922]	
Sedentary time (min/day)	557 [338; 591]	667 [638; 724]	
Prolonged sedentary time^b^ (min/day)	220 [72; 342]	304 [162; 435]	
Number of sedentary bouts per day	12 [4; 15]	16 [11; 20]	
LPA time (min/day)	152 [88; 274]	131 [52; 239]	
MVPA time (min/day)	45 [13; 151]	39 [20; 53]	
**Relative to total wear time (%)**			
Overall sedentary time	75 [51; 85]	80 [70; 90]	0.14
Prolonged sedentary time	30 [8; 53]	34 [18; 62]	0.14
LPA time	20 [13; 32]	15 [7; 26]	0.07*
MVPA time	6 [2; 18]	4 [3; 6]	0.27

*LPA, light physical activity; MVPA, moderate-to-vigorous physical activity*.

a *Differences in baseline blood levels and accelerometer-derived data (i.e. data relative to wear time) were tested using Wilcoxon Signed Rank Tests*.

b *Prolonged sedentary time was defined as the time spent sedentary accumulated in bouts of ≥10 min*.

# *Due to technical problems, valid accelerometer data were not available for one participant during the normal days, for one participant during the prolonged sedentary days and for one participant during both the normal days and the prolonged sedentary days. In total, valid accelerometer data for both the normal days and the prolonged sedentary days was available for four participants*.

*Significantly higher during prolonged sedentary days when compared to normal days.

Table 1 presents overall and prolonged sedentary time, LPA time, and MVPA time during 6 normal days before the experiment and during the 6 prolonged sedentary days. Unfortunately, due to technical problems with the accelerometers, only four participants had valid data for both the 6 normal days as well as the 6 prolonged sedentary days. Strikingly, during the 6 normal days participant's median sedentary time was quite high: 9.3 h/day sedentary, of which 3.7 h/day prolonged. During the prolonged sedentary days, participants spent 14.7 h/day sedentary, of which 5.1 h/day prolonged, thereby meeting the requests of interrupted and uninterrupted sedentary time. After adjusting for wear time, overall sedentary time, and prolonged sedentary time were slightly but not significantly higher during the prolonged sedentary days compared to the normal days. LPA was 5% lower during the 6 prolonged sedentary days compared to the normal days (i.e., 5% of wear time), whereas MVPA time was similar (Table 1).

**Figure 2:**
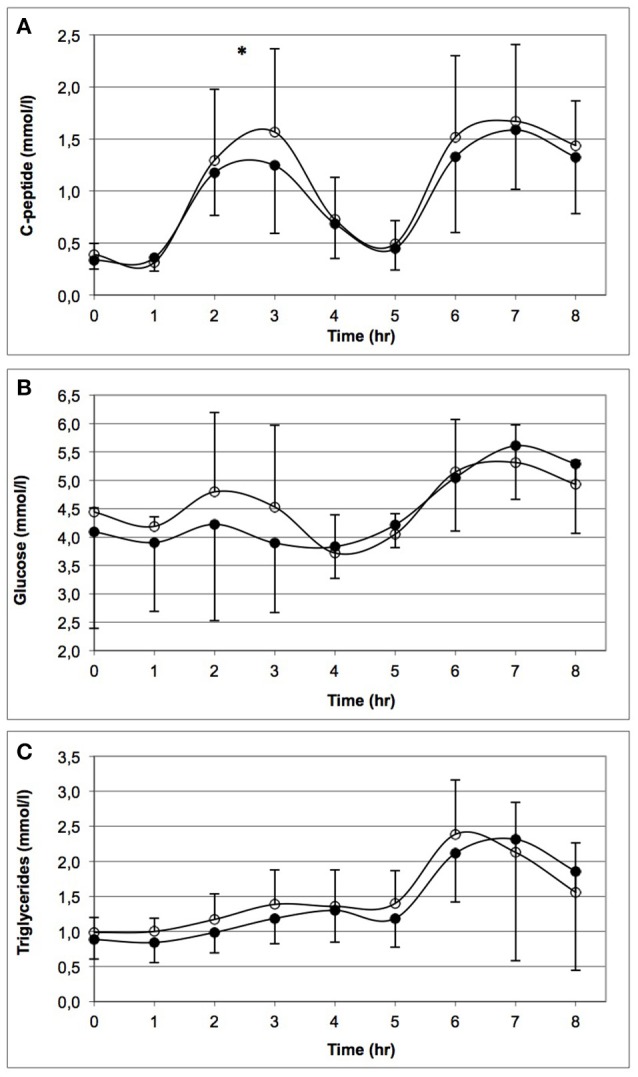
**Levels of C-peptide (A)**, glucose **(B)**, and triglycerides **(C)** throughout 1 day of prolonged sitting before (closed circles) and after (open circles) 6 prolonged sedentary days. ^*^Indicates significant higher levels of C-peptide for the second laboratory-sitting day compared to the first day, i.e., following the 6 prolonged sedentary days. Note that the baseline measurements are slightly different between time points (*t* = 0 and 1) and laboratory days. Standardized high fat mixed meals were consumed at *t* = 1 and 5.

Figure 2 demonstrates the levels of C-peptide, glucose, and triglycerides throughout 1 day of prolonged sedentary time before (closed circles) and after (open circles) 6 prolonged sedentary days. GEE analysis for the 7-h period, including the response to both standardized meals revealed a significant higher postprandial C-peptide levels during the second laboratory day following the 6 prolonged sedentary days compared to the first laboratory day (B = 0.11, 90% CI = [0.002; 0.22]; Table 2). Median C-peptide AUC and iAUC for the 7-h period were 7 and 16% larger after 6 prolonged sedentary days (Table 3). Glucose and triglycerides levels were not significantly different between the 2 laboratory days. Results for the first 4-h period (including the response to the first standardized meal only) were similar (Table 2).

**Table 2:** **Difference (unstandardized regression coefficient (B) and 90% CI) in cardiometabolic risk factors between laboratory sitting day at baseline and following 6 prolonged sedentary days**.

	**B [90% CI]**	
	**7-h period**	**4-h period**
Glucose (mmol/l)	0.08 [−0.07; 0.24]	0.10 [−0.10; 0.30]
C-peptide (mmol/l)	0.11 [0.002; 0.22]^*^	0.10 [0.01; 0.18]^*^
Triglycerides (mmol/l)	0.08 [−0.21; 0.36]	0.16 [−0.02; 0.35]

* *Significantly higher postprandial C-peptide during the second laboratory-sitting day following 6 prolonged sedentary days compared to the first laboratory-sitting day*.

*Note that a positive B indicates a higher blood level for the second laboratory-sitting day following 6 prolonged sedentary days compared to the first laboratory day*.

**Table 3:** **Postprandial plasma glucose, C-peptide, and triglyceride area under the curve (median [min; max]) before and after 6 prolonged sedentary days**.

	**Time (h)**	**1st laboratory day**	**2nd laboratory day**
**AUC**			
Glucose	7	31.5 [21.9; 39.1]	31.4 [26.0; 39.0]
	4	16.4 [10.9; 21.6]	16.1 [14.4; 23.5]
C-peptide	7	7.1 [3.8; 11.4]	7.7 [4.0; 13.6]
	4	3.5 [1.9; 5.2]	3.9 [1.6; 6.8]
Triglyceride	7	9.9 [6.3; 15.8]	10.2 [7.5; 15.5]
	4	4.3 [2.8; 7.3]	4.5 [3.8; 7.6]
**iAUC**			
Glucose	7	2.3 [0.1; 4.7]	2.2 [0.1; 7.8]
	4	0.3 [0; 1.8]	0.2 [0; 5.1]
C-peptide	7	4.5 [2.0; 8.0]	4.9 [2.3; 10.3]
	4	1.5 [0.7; 2.9]	2.2 [0.6; 4.5]
Triglyceride	7	4.0 [2.0; 8.8]	3.8 [1.0; 8.3]
	4	1.2 [0.3; 1.6]	0.6 [0; 2.7]

*AUC, area under the curve; iAUC, incremental area under the curve*.

## Discussion

This pilot study explored the postprandial effects of multiple days of prolonged sedentary time in free-living conditions on metabolic risk factors in healthy young men. During the execution of this study, we encountered a number of important implications for future studies. Therefore, we first discuss the findings of our pilot study on the metabolic risk factors in healthy young men. Subsequently, we discuss the lessons we have learned from this study and recommendations for future studies.

### Pilot findings on metabolic risk factors

Despite the relatively small increase in interrupted and uninterrupted sedentary time, we found higher postprandial levels of C-peptide following 6 prolonged sedentary days, when compared to baseline. The higher levels of postprandial C-peptide during several prolonged sedentary days is in contrast with previous studies in middle-aged, overweight/obese adults (Thorp et al., 2014; Larsen et al., 2015), but in line with a previous study in free-living, healthy, young adults (Lyden et al., 2015). Maintenance of normal fasting and postprandial glucose levels depend on the ability to create an adequate insulin response to a meal. The loss of local contractile stimulation in weight-bearing muscles may lead to reduced triglycerides uptake, through the suppression of skeletal muscle lipoprotein lipase (LPL) activity (Bey and Hamilton, 2003; Hamilton et al., 2004), as well as reduced glucose uptake. The contrasting findings regarding the potential unfavorable effects of consecutive prolonged sedentary on postprandial (endogenous) insulin may be explained by the number of prolonged sedentary days. Thorp et al. (2014) and Larsen et al. (2015) examined postprandial effects after 3 prolonged sedentary days, whereas Lyden et al. (2015) and this study examined postprandial effects after seven and 6 prolonged sedentary days, respectively. The potential adverse effects of prolonged sedentary time may only emerge when sustained for a number of consecutive days (e.g., six or more). Possibly, a healthy lifestyle may hold off unfavorable adverse effects of prolonged sedentary time, e.g., by sleeping adequately (Morselli et al., 2010). Future studies should examine this hypothesis. Another explanation may be that the participants in our study and the study of Lyden et al. (2015) were healthy and physically active adults, while participants in the studies of Thorp et al. (2014) and Larsen et al. (2015) were overweight/obese and low active adults.

Our pilot study demonstrated that the postprandial level of C-peptide was 0.11 mmol/l higher after 6 days of increased sedentary time. The clinical importance of this finding needs further study, by prospectively examining the effects of prolonged sedentary time on metabolic risk factors and the incidence of type 2 diabetes and cardiovascular disease.

Although the design of the study of Lyden et al. (2015) is similar to the present study, the difference in postprandial response measurement (i.e., 2-h oral glucose tolerance test vs. 4- and 7-h meal response in our study, and insulin vs. C-peptide) hampers comparison between the two studies. Moreover, in the study of Lyden et al. (2015) participants spent more time sedentary during the increased free-living sedentary days (i.e., 15 vs. 5% in the present study). The 7 and 16% larger 7-h C-peptide AUC and iAUC, respectively, may have a substantial detrimental effect on cardiometabolic risk, especially when considering the small increase in sedentary time. A *post-hoc* sample size calculation based on the present findings revealed that 64 participants are needed to detect a 0.11 mmol higher postprandial C-peptide level after 6 prolonged sedentary days, using a significance level of 0.05 and a power of 80%.

In line with previous studies examining multiple prolonged sedentary days (Thorp et al., 2014; Larsen et al., 2015; Lyden et al., 2015) we found no significant difference in postprandial glucose levels. As proposed by Lyden et al. (2015), the lack of changes in postprandial glucose, as opposed to increases in postprandial insulin, may indicate the importance of insulin action in the development of cardiometabolic ill-health induced by prolonged sitting.

We found no significant difference in postprandial triglyceride levels following the 6 consecutive prolonged sedentary days. A postprandial increase in triglycerides has been related to decreased insulin sensitivity (Axelsen et al., 1999; Annuzzi et al., 2004; Madhu et al., 2008), which is not expected in young and healthy subjects. However, when analyzing only the first 4 h of the laboratory days, including the response of the first standardized meal only, the effect size for triglyceride levels doubled (Table 2). This might be caused by the difference in consistency of the standardized meals, i.e., the first meal was liquid, whereas the second meal was solid. Since liquid food is more rapidly emptied from the stomach than solid food (Read and Houghton, 1989), the first meal might have raised blood lipids to a higher extent than the second meal.

### Lessons learned and recommendations

Our first recommendation is that future experimental studies should examine the potential adverse metabolic health effects of sedentary patterns that are more realistic in real life. Participants were slightly but not significantly more sedentary during the 6 prolonged sedentary days when compared to the normal days, both overall (i.e., 5% of wear time) and prolonged (i.e., 4% of wear time), and spent slightly less time on LPA (i.e., 5% of wear time). The relatively small increase in interrupted and uninterrupted sedentary time in our study may indicate that in free-living conditions it is difficult for young, physically active males to increase their sedentary time substantially. Thus, patterns of 6–8 h of prolonged sitting, as examined in previous experimental laboratory studies on the adverse health effects of prolonged sitting, are rare in young and healthy males. Additionally, we recommend future studies to monitor a full day including sleep, as adequate sleep may influence participants' metabolism (Morselli et al., 2010).

Secondly, we recommend future studies examining potential adverse effects of increased sedentary time to check participants' normal PA and sedentary behavior using objective measures, i.e., as a pre-study screening, to make sure the requested increase in prolonged sedentary time is indeed a substantial increase compared to normal weeks. Additionally, when participants know their normal prolonged sedentary time it may be more feasible for them to reach a certain sedentary time prescription. In the present study participants were screened by dialogue to check whether they spent <2 h on prolonged sedentary time on a regular day. Baseline accelerometer data demonstrated that the healthy young men in our study spent on average 9.3 h/day sedentary of which 3.7 h/day prolonged, indicating that they underestimated their normal prolonged sedentary time during screening. As a consequence of the considerable amount of their baseline sedentary time, participants had limited opportunity to further increase their sedentary time substantially.

Another recommendation for future studies is to examine the longer-term health effects of both overall and prolonged sitting time in free-living conditions, thereby including measures that can differentiate between lying, sitting and standing (e.g., ActivPAL). Hip-worn accelerometers are widely used to measure sedentary time yet they cannot distinguish between various postures (i.e., lying, sitting, standing). The limitation that accelerometers are not accurate enough for assessing sedentary time may be another explanation for the relatively small increase in interrupted and uninterrupted sedentary time in our study.

Next, we recommend future intervention studies that targeting to increase LPA time may be a potential effective strategy when aiming to reduce sedentary time. Our study demonstrated that the small decrease in LPA time coincided the small increase in sedentary time may, indicating that participants substituted their LPA time with sedentary time.

### Strengths and limitations

Strengths of this study include the hourly blood collection and the focus on both glucose and lipid metabolism. The inclusion of healthy young men additionally strengthens our study, since the influence of confounding of disease processing (i.e., obesity, type 2 diabetes), and menstrual cycle was eliminated. The “real life” setting (i.e., imposing days of predominantly sitting) further strengthens our study. A limitation is the small sample size. Moreover, due to incomplete accelerometer data we cannot confirm that all participants actually increased their (uninterrupted) sedentary time. Finally, we did not standardize dietary intake during the 6 prolonged sedentary days. However, as participants consumed a standardized meal on the evening before each laboratory days, we expect this influence to be minimal. Future studies should examine this.

## Conclusion

We conclude that multiple days of prolonged sedentary time may have an unfavorable effect on postprandial C-peptide levels, even in healthy young men. Acute metabolic effects of prolonged sedentary time may accumulate when sustained for multiple days, and therefore needs further study. Besides hypothesis testing experimental studies, we recommend future studies to examine metabolic effects of sedentary patterns that fit real life conditions.

## Author contributions

TA conceived and designed the study, collected, analyzed and interpreted the data, and wrote the manuscript. JR, ES, and MC were involved in conceiving and designing the study, data interpretation, and drafting the manuscript. All authors read and approved the final manuscript.

## Funding

The contributions of TA and MC were funded by the Netherlands Organization for Health Research and Development (ZonMw Project nr. 91211057).

### Conflict of interest statement

The authors declare that the research was conducted in the absence of any commercial or financial relationships that could be construed as a potential conflict of interest.
